# Handheld dermatoscopy as an easy-to-use capillaroscopic instrument in rheumatoid arthritis: a cross-sectional study

**DOI:** 10.3906/sag-2006-6

**Published:** 2020-10-22

**Authors:** Ömer Faruk ELMAS, Mehmet OKÇU, Abdullah DEMİRBAŞ, Necmettin AKDENİZ

**Affiliations:** 1 Department of Dermatology and Veneorology, Faculty of Medicine, Ahi Evran University, Kırşehir Turkey; 2 Department of Physical Therapy and Rehabilitation, Faculty of Medicine Ahi Evran University, Kırşehir Turkey; 3 Department of Dermatology and Veneorology, Konya Numune State Hospital, Konya Turkey; 4 Department of Dermatology and Veneorology, Faculty of Medicine, İstanbul Medeniyet University, İstanbul Turkey

**Keywords:** Dermatoscopy, handheld dermatoscopy, nailfold dermatoscopy, nailfold capillaroscopy, rheumatoid arthritis

## Abstract

**Background/aim:**

Nailfold video capillaroscopy is considered as a reliable method for evaluating peripheral microangiopathy in rheumatologic diseases. In this study, we aimed to demonstrate the utility of handheld dermatoscopy as an easy-to-use nailfold capillaroscopic instrument in patients with rheumatoid arthritis.

**Materials and methods:**

This cross-sectional study included patients with rheumatoid arthritis and healthy subjects. A handheld dermatoscopic examination of proximal nail fold was performed in each subject. The possible correlation of capillaroscopic findings with disease activity was evaluated using the disease activity score 28 (DAS28).

**Results:**

A total of 59 patients with rheumatoid arthritis and 60 healthy subjects were enrolled in the study. The presence of capillary enlargement, avascular areas, capillary deformities, and capillary vascular anomalies in the group of patients showed a statistically significant difference when compared with the healthy subjects. No correlation was found between the nail fold capillaroscopic findings and DAS28 score.

**Conclusion:**

Hand-held dermatoscopy seems to be a useful technique in the evaluation of nail fold capillary changes. We suggest that in patients with rheumatoid arthritis, when capillaroscopic examination is needed, it can be evaluated using handheld dermatoscopy. Selected patients who showed findings using this method can be further examined with classical capillaroscopy to obtain more quantitative data.

## 1. Introduction

Rheumatoid arthritis (RA) is characterized by inflammation in the synovial joints with a potential for multisystemic involvement [1–4]. Pulmonary, cardiac, neurological, ocular, hematological, and renal involvement can be observed [5].

Vascular structural change plays an important role in the pathophysiology of RA. In RA, the microvascular damage occurs as a result of increased capillary permeability and changes in the extracellular matrix, and endothelial connections in dermal papillae. Abnormal capillary shapes and structures are the morphological indicators of microvascular damage [6–7].

Nailfold videocapillaroscopy (NVC) is a safe and reliable technique for analyzing microcirculation of dermal papillae in nailfold [8]. Nailfold videocapillaroscopic patterns of systemic sclerosis (SSC), systemic lupus erythematosus (SLE) and dermatomyositis-polymyositis (DM-PM) have been well-described [9–10]. However, there are a few studies investigating the NVC findings in patients with RA. Recent studies showed that nailfold dermatoscopy (ND) is comparable to NVC in the assessment of nailfold capillaries in patients with systemic sclerosis spectrum disorders [11]. To the best of our knowledge, however, no studies focusing on the use of ND in RA exist in the relevant literature [12–13].

In this study, we aimed to demonstrate the utility of polarized handheld dermatoscopy as a nailfold capillaroscopic instrument in patients with RA.

## 2. Materials and method

### 2.1. Subjects

This cross-sectional study included patients with rheumatoid arthritis in healthy patients, matched according to age and sex. The subjects were selected from the patients with RA who were admitted to the outpatient department of physical medicine and rehabilitation in a university hospital between January 2019 and January 2020. All of the subjects with RA fulfilled the classification criteria of American Rheumatology Association/European Rheumatism Association RA classification criteria [14].

Patients with accompanying rheumatological diseases, diabetes mellitus, hypertension, primary and secondary Raynaud’s phenomenon, acrocyanosis, cardiovascular disease, and coagulopathy were excluded. All subjects who had nail care in the last month or those with a habit of nail eating were also ruled out. 

Demographic, clinical, and laboratory parameters collected from each patient included age, sex, disease duration, number of tender joints, number of swollen joints, patient’s self-assessment of disease activity, c-reactive protein levels, and erythrocyte sedimentation rate. 

Disease activity was evaluated using the disease activity score 28 (DAS28). Tender joint count, swollen joint count, patients’ global health assessment and erythrocyte sedimentation rate were the parameters used to calculate the DAS 28 score. DAS28-ESR <2.6 was considered as “remission”. High disease activity relates to DAS28 more than 5.1 and moderate activity relates to DAS28 between 3.2 and 5.1. Low disease activity is regarded in the range of 2.6 to 3.2 [15].

### 2.2. Nailfold dermatoscopic capillaroscopy

The nailfold dermatoscopic examination was performed using a 10­fold polarized handheld dermatoscope (Dermlite DL4, 3gen Inc, CA, USA). The dermatoscopic images were obtained using an attached high-resolution mobile camera phone with 2× optical zoom (iPhone 7 plus, Apple Inc, CA, USA). Thus, a total of 20× magnification was achieved. All ten fingers of the subjects were examined using isopropyl alcohol for clear visualization of the capillary structures. During the procedure, patients’ hands were held at the level of the heart and pressure on the nail fold was especially avoided, not to distort capillary structures. 

The capillary morphology was evaluated using the Maricq criteria, which was rearranged by Bergman et al. [16–17]. Megacapillaries (elongated/enlarged capillaries), avascular areas (at least two consecutive avascular capillary areas), microhemorrhages (bleeding at a minimum of two points around a single capillary), capillary deformities (tortuosity/branching/angulation), and capillary vascular anomalies (the presence of at least two morphological features in at least two nail folds) were the evaluated capillaroscopic parameters for each subject. An experienced dermatologist evaluated the dermatoscopic images obtained in terms of the presence of aforementioned features

### 2.3. Statistical analysis

Descriptive values of the data obtained were calculated as mean, standard deviation, median, minimum, maximum, interquartile range, number, and percentage. The suitability of the data to normal distribution was evaluated with the Kolmogorov–Smirnov test. The difference between the groups for the numerical variables was analyzed with the Mann–Whitney U test and for the categorical variables with the chi-square test. The difference of DAS28 score in categorical variables was tested with the Mann–Whitney U test, and the relationship with activity was tested with the Fisher–Freeman–Halton test. The statistical significance level was set as P < 0.05. SPSS Windows version 24.0 package software (SPSS Inc.; Chicago, IL, U.S.A) was used for statistical analysis.

### 2.4. Ethical approval 

All the procedures followed the Helsinki declaration and the project was approved by the Institutional Review Board (decision date and number: 2018-22/185). Written informed consent was also obtained from all the subjects.

## 3. Results

**Figure 1:**
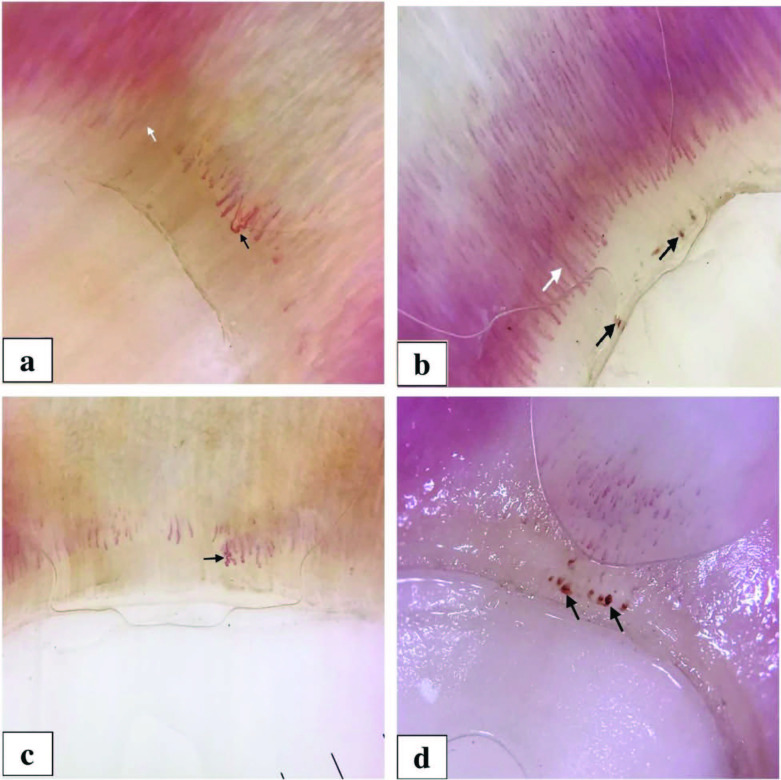
a. Megacapillary structures (enlarged capillaries) (black arrow), avascular area (white arrow). b. Megacapillary structures (elongated capillaries) (white arrow), microhemorrhagic foci (black arrow). c. Capillary deformity (tortuous, branched and angulated capillaries) (arrow) d. Microhemorrhagic foci (arrows).

A total of 59 patients with RA and 60 healthy subjects were enrolled in the study. The mean ages of the patients and controls were 56.28 ± 13.66 and 57.38 ± 13.37 years, respectively. The majority of the patients were female (n = 45) and the mean disease duration was 9.842 ± 10.968 years. The mean DAS28 score was 2.74 ± 1.15. The presence of megacapillaries (Figures 1a and 1b), avascular areas (Figure 1a), capillary deformities (Figure 1c), and capillary vascular anomalies in the patients group showed a statistically significant difference when compared with the healthy subjects (P = 0.004, P = 0.001, P = 0.001, and P = 0.001, respectively). There was no statistically significant difference between the two groups in terms of microhemorrhage (Figures 1b and 1d) (P = 0.660). There was no statistically significant relationship between DAS28 scores and the presence of nailfold capillaroscopic findings. Distribution of the sex, age and nailfold capillaroscopic features in the patients and healthy subjects were given in Table. 

**Table:** Distribution of the sex, age, and nailfold dermatoscopic features in patients and healthy subjects.

Data	Control (n = 60)	Patient (n = 59)	P-value
Sex (male /female)	14a / 46a	14a / 45a	0.959a
Age (years, Mean ± SD)	57.383 ± 13.378	56.281 ± 13.669	0.690
Megacapillary [n (%)]	0a (0.0)	22b (43.1)	0.001a
Capillary Deformity [n (%)]	10a (16.7)	21b (41.2)	0.004a
Microhemorrhage [n (%)]	2a (3.3)	3a (5.9)	0.660b
Avascular areas [n (%)]	0a (0.0)	12b (23.5)	0.001a
Capillary vascular anomaly [n (%)]	0a (0.0)	27b (52.9)	0.001a

a: Pearson chi-square, b: Fisher’s exact test, SD: Standard deviation, P < 0.05 is defined as statistically significant and shown in bold.

## 4. Discussion

RA is a chronic, progressive, inflammatory autoimmune disease that may have extensive systemic findings [3]. In recent studies, endothelial cells have been shown to play a key role in inflammatory events in cases of RA showing systemic findings. The level of angiogenesis mediators such as vascular endothelial growth factor, interleukin-8, tumor necrosis factor-alpha, and endothelin-1 are increased in these patients [18,19]. The angiogenesis mediators have been shown to induce systemic vasculitis, which causes severe complications and shortened lifespan in patients with RA [20–21]. Changed vascular environment is a notable feature of RA’s pathogenesis. Functional and morphological injuries of microcirculation have been described in patients with RA. These injuries are reported to be the outcome of capillary permeability impairments and changes in endothelial connections with the extracellular matrix, which induces strange capillary configurations and structures in the dermal papillae. Nailfold capillaroscopy can easily identify peripheral microangiopathy, even in the early stages of the disease. Nailfold capillaroscopy is considered a simple, noninvasive, cheap, and sensitive method for the morphological evaluation of microcirculation in patients with systemic inflammatory disorders [8]. In the present study, 52.54% of RA patients showed at least one nailfold capillaroscopic finding. Compared to the control group, the incidence of megacapillary, capillary deformity, avascular areas and capillary vascular anomalies was statistically significantly higher in RA patients.

The reliability of the NVC method in systemic sclerosis and Raynaud’s phenomenon has been demonstrated in several studies [22,23]. Moreover, it has been shown that ND can provide comparable results to NFC in the same diseases [8]. However, there is no study investigating the reliability of NVC and ND in patients with RA. In this study, ND findings in RA patients were compared with those of healthy controls and meaningful results were obtained. Our study showed that ND may be a reliable capillaroscopic instrument in RA, however, reliability studies comparing NVC and ND should provide more conclusive results. No specific NVC pattern has been identified for RA in the relevant studies so far [7]. In a recent study, 45.7% of 201 patients with RA were noted to have NVC findings. In our study, similarly, 52.54% of the patients showed at least one abnormal nailfold dermatoscopic feature. In a series of 430 patients with RA, the most common NVC finding was tortuosity followed by angiogenesis, enlarged loops, and microhemorrhage [24]. Altomonte et al. [25] and Lin et al. [26] also revealed that tortuosity and enlarged loops are the main NVC findings in patients with RA. In our study, similarly, the most common ND features were megacapillaries (enlarged loops) and capillary deformities (tortuosity, branching or angulation). Avascular areas were found in 23.5% of the patients while none of the controls showed this finding. Avascular areas were more frequent in our study when compared to the study of Rajaei et al. in which they were found in only 0.6% of 359 patients with RA [24]. Scleroderma-like capillaroscopic patterns in patients with RA were found in a range of 0.49% to 20.9% in different studies [22,24,25,27]. In our study none of the subjects showed scleroderma-like patterns.

When patients with RA who had nailfold capillary changes (52.54%) are compared with the ones whose dermatoscopic findings are normal (47.45%), no statistically significant difference was observed in terms of the DAS28 score (P > 0.05). Sag at al. also did not find a statistically significant difference between the patients with and without abnormal nailfold capillary structures in terms of the severity of the disease [27].

In the study by Errichettiet al., in which nail fold and elbow dermatoscopic features of patients with early psoriatic arthritis and early RA were investigated, nail fold dermatoscopic features of 12 patients with RA included “fish school-like” vascular pattern and irregular/ramified, blurry, purple vessels [28]. In another study conducted by Zabotti at al. the only nail fold dermatoscopic feature in patients with RA was “dotted” vessels which were observed in 7 out of 42 early cases of RA [29]. Both studies included only early cases of RA [28,29]. 

In our study, we investigated nail fold dermatoscopic findings in detail in patients with RA with different severities and compared the results with healthy controls. We also investigated the possible association of the findings identified with the disease severity. These two points were the major advantages of this study. On the other hand, the relatively small size of the study population and lack of the morphometric measurement of identified capillary structures were the main limitations.

To conclude, hand-held dermatoscopy seems to be a useful and easy technique in the evaluation of capillary nailfold morphological change. Patients with RA can be evaluated with a hand-held dermatoscope when nailfold capillaroscopic examination is needed. Selected patients who have remarkable findings using this method can be examined in detail with classical capillaroscopy in order to obtain more quantitative data.

## References

[ref1] (2008). Rheumatoid arthritis and cardiovascular disease. Current Atherosclerosis Reports.

[ref2] (2011). Early diagnosis and treatment outcomes. Caspian Journal of Internal Medicine.

[ref3] (2009). The vasculature in rheumatoid arthritis: cause or consequence?. International Journal of Experimental Pathology.

[ref4] (2007). Extra-articular manifestations and complications of rheumatoid arthritis. Best Practice & Research Clinical Rheumatology.

[ref5] (2011). Demographic, clinical, and serological features of Turkish patients with rheumatoid arthritis: evaluation of 165 patients. Clinical Rheumatology.

[ref6] (1981). Capillary microscopy of the nailfold in psoriatic and rheumatoid arthritis. Scandinavian Journal of Rheumatology.

[ref7] (1994). Increased capillary permeability in systemic rheumatoid vasculitis: detection by dynamic fluorescence nailfold videomicroscopy. The Journal of Rheumatology.

[ref8] (2013). How to perform and interpret capillaroscopy. Best Practice & Research Clinical Rheumatology.

[ref9] (2013). classification criteria for systemic sclerosis: an American College of Rheumatology/European League against Rheumatism collaborative initiative. Arthritis & Rheumatism.

[ref10] (2008). the nailfold microvascular changes during the capillaroscopic analysis in systemic sclerosis patients. Annals of the Rheumatic Diseases.

[ref11] (2018). The assessment of nailfold capillaries: comparison of dermoscopy and nailfold videocapillaroscopy. Rheumatology.

[ref12] (2013). Capillaroscopy in psoriatic and rheumatoid arthritis: a useful tool for differential diagnosis. Arthritis.

[ref13] (2012). Comparison of qualitative and quantitative analysis of capillaroscopic findings in patients with rheumatic diseases. Rheumatology International.

[ref14] (2010). rheumatoid arthritis classification criteria: an American College of Rheumatology/European League Against Rheumatism collaborative initiative. Arthritis & Rheumatism.

[ref15] (1995). Modified disease activity scores that include twenty‐eight‐joint counts development and validation in a prospective longitudinal study of patients with rheumatoid arthritis. Arthritis & Rheumatism: Official Journal of the American College of Rheumatology.

[ref16] (2003). The handheld dermatoscope as a nail-fold capillaroscopic instrument. Archives of Dermatology.

[ref17] (2019). Dermatoscopic assessment of nailfold capillary abnormalities in Behçet’s disease and correlation of the findings with disease activity and severity. Dermatologica Sinica.

[ref18] (2011). The pathogenesis of rheumatoid arthritis. New England Journal of Medicine.

[ref19] (2006). A study on vascular endothelial growth factor and endothelin-1 in patients with extra-articular involvement of rheumatoid arthritis. Clinical Rheumatology.

[ref20] (1986). Survival, prognosis, and causes of death in rheumatoid arthritis. Arthritis & Rheumatism: Official Journal of the American College of Rheumatology.

[ref21] (1981). Systemic rheumatoid vasculitis: a clinical and laboratory study of 50 cases. Medicine (Baltimore).

[ref22] (2013). Reliability of widefieldnailfoldcapillaroscopyand video capillaroscopy in theassessment of patientswithRaynaud’sphenomenon. ArthritisCareRes (Hoboken).

[ref23] (2017). Intra-andinter-observerreliability of nailfoldvideocapillaroscopy - A possibleoutcomemeasureforsystemicsclerosis-related microangiopathy. Microvasc Res.

[ref24] (2017). Nailfold capillaroscopy in 430 patients with rheumatoid arthritis. Caspian Journal of Internal Medicine.

[ref25] (1995). Microvascular capillaroscopic abnormalities in rheumatoid arthritis patients. Clinical and Experimental Rheumatology.

[ref26] (2009). Clinical applications of nailfold capillaroscopy in different rheumatic diseases. J Intern Med Taiwan.

[ref27] (2017). Nailfold videocapillaroscopy results in patients with rheumatoid arthritis. Clinical Rheumatology.

[ref28] (2016). Dermoscopy of nail fold and elbow in the differential diagnosis of early psoriatic arthritis sine psoriasis and early rheumatoid arthritis. The Journal of Dermatology.

[ref29] (2018). Early psoriatic arthritis versus early seronegative rheumatoid arthritis: role of dermoscopy combined with ultrasonography for differential diagnosis. The Journal of Rheumatology.

